# Plausible subjective experience versus fallible corroborative evidence: The formulation of insanity in Nigerian criminal courts

**DOI:** 10.3389/fpsyt.2023.1084773

**Published:** 2023-04-19

**Authors:** Adegboyega Ogunwale, Letitia Pienaar, Oluwaseun Oluwaranti

**Affiliations:** ^1^Neuropsychiatric Hospital Aro, Abeokuta, Nigeria; ^2^Forensic & Neurodevelopmental Sciences Department, Institute of Psychiatry, Psychology & Neuroscience, King's College London, London, United Kingdom; ^3^Department of Criminal and Procedural Law, College of Law, University of South Africa, Pretoria, South Africa; ^4^Nottinghamshire Healthcare NHS Foundation Trust, Nottingham, United Kingdom

**Keywords:** subjective, formulation, Nigeria, insanity defence, corroborative evidence, phenomenology

## Abstract

Insanity as a defence against criminal conduct has been known since antiquity. Going through significant reformulations across centuries, different jurisdictions across the globe, including Nigeria, have come to adopt various strains of the insanity defence, with the presence of mental disorder being the causative mechanism of the crime as their central theme. A critical ingredient in the Nigerian insanity plea is the presence of ‘mental disease’ or ‘natural mental infirmity’ as the basis for the lack of capacity in certain cognitive and behavioural domains resulting in the offence. Mental disorders, which are the biomedical formulations of this critical legal constituent are primarily subjective experiences with variable objective features. Using illustrative cases based on psycho-legal formulation as well as reform-oriented and fundamental legal research, it is shown that Nigerian courts have held that claims of insanity based on the accused person’s evidence alone should be regarded as “suspect” and not to be “taken seriously.” Thus, Nigerian judicial opinions rely on non-expert accounts of defendants’ apparent behavioural abnormalities and reported familial vulnerability to mental illness, amongst other facts while conventionally discountenancing the defendants’ plausible phenomenological experiences validated by expert psychiatric opinion in reaching a conclusion of legal insanity. While legal positivism would be supportive of the prevailing judicial attitude in entrenching the validity of the disposition in its tenuous precedential utility, legal realism invites the proponents of justice and fairness to interrogate the merit of such preferential views which are not supported by scientific evidence or philosophical reasoning. This paper argues that disregarding the subjective experience of the defendant, particularly in the presence of sustainable expert opinion when it stands unrebutted is not in the interest of justice. This judicial posturing towards mentally abnormal offenders should be reformed on the basis of current multidisciplinary knowledge. Learning from the South African legislation, formalising the involvement of mental health professionals in insanity plea cases, ensures that courts are guided by professional opinion and offers a model for reform.

## 1. Introduction

Since ancient times, the defence of insanity against criminal behaviour has been recognised (1–3). The M’Naghten’s rule, established in English law in 1843 (M’Naghten’s case, 1843; R v. M’Naghten, 1843), served as a precursor of contemporary tests of legal insanity in several areas of the world. Given their relative lack of mens rea, persons who are mentally ill are not to be held criminally accountable for their unlawful behaviour (2, 4). Due in significant part to its colonial background, Nigeria, a former British colony and a lower middle-income Commonwealth nation in West Africa, has strong ties to the British common law tradition (Laws of the Federation of Nigeria, 2004c).

Thus, the M’Naghten’s rules’ elements were essentially replicated in the Nigerian insanity defence until 1948, when a volitional prong was added by judicial pronouncement (Laws of the Federation of Nigeria, 2004a; Rex v Ashigifuwo, 1948; Rex v Omoni, 1949). The M’Naghten’s rule has been criticised in the legal literature for having an excessively cognitivistic slant that ignores the importance of volition in human conduct (5). The extension of the provisions of the insanity plea to include the lack of capacity to control one’s action represented an attempt at recognising conative defects which may accompany mental disorder (5, 6). For instance, studies have shown that patients with schizophrenia may experience “passivity” – the control of their own actions or thoughts by perceived external agents (7, 8). Under such conditions, they may lack the sense of agency necessary for the exercise of self-control or conforming their conduct to the requirements of the law (9). Other mental disorders such as intermittent explosive disorder, kleptomania and pyromania (10) may also be implicated as conditions meeting the legal criterion of lack of volition. It is crucial to note however that in the Nigerian legal formulation, crossing the threshold of such a defence will depend on the circumstances of the case as well as the previous conduct of the defendant, and not merely a subjective assertion of inability to control oneself (Rex v Omoni, 1949).

Furthermore, the Supreme Court of Nigeria has maintained a view that insanity is to be determined in the legal sense and is an issue of fact that should be decided by the courts rather than physicians in the practical formulation of insanity in Nigerian courts (11). It is also to be viewed as dependant on the defendant’s prior and present actions, and its burden of proof is one based on the preponderance of the evidence or the balance of probabilities (Emeryl v State, 1973; Madjemu v State, 2001; Rex v Ashigifuwo, 1948). In Madjemu, the Supreme Court also emphasised the following “criteria” as important to the assessment of insanity under the law:

- Evidence as to the past history of the accused.
- Evidence as to the conduct of the accused immediately preceding the killing of the deceased.
- Evidence of prison officials who had custody of the accused before and during his trial.
- Evidence of medical officers who examined the accused.
- Evidence of relatives about the general behaviour of the accused and the reputation he enjoyed for sanity or insanity in the neighbourhood.
- Evidence showing the insanity runs in the family of the accused.
- Other facts which will help to discharge the burden of proof.

A crucial evidentiary formulation was added by the Supreme court in Guobadia v State (2004) when it held that: “evidence of insanity tendered by an accused person himself is suspect and is not usually taken seriously.”

A number of reasons may be adduced for this judicial position. First, the court may be wary of deception on the part of the accused, given the seriousness of the crime of murder which is the usual impetus for the insanity plea (12). A recent analysis of all cases involving the insanity plea reported in Nigerian law reports revealed that 100% of them were homicide cases (12) although the study only included reported appeal cases. Malingering, which is essentially the faking of physical/mental symptoms occurs among criminal defendants (13–15). Second, the court must assert itself as the final arbiter on legal insanity based on its competence as a trier of facts. To avoid perverse outcomes, it must adhere to consistency in its logical reasoning across all components of a case. Third, in theory, the concept of adverse inference would seem to permit the court to form a negative opinion of a defendant’s claim as may be found across jurisdictions (16, 17). Adverse inference refers to situations in which liability could be inferred from silence or failure to cooperate with producing evidence. Granted that the rule of adverse inference usually applies to defendants who choose to keep silent in the face of indictment, the principle is one that could potentially explain the attitude of Nigerian courts to uncorroborated subjective claims of insanity. However, this is not supported by the criminal procedure legislation (Laws of the Federation of Nigeria, 2004b), evidentiary rules (Evidence Act, 2011) or the constitution (Constitution of the Federal Republic of Nigeria, 1999). Finally, it may be argued that uncorroborated personal assertions of insanity would amount to a waste of the court’s precious time and other resources by allowing defendants to take the system on a wild-goose chase.

On the other hand, this judicial position has inherent problems. First, it appears arbitrary. Arbitrary, in general, has been defined as “not supported by fair, solid, and substantial cause, and without reason given” while arbitrary discretion has been described as: “a decision that is made wrongfully, possibly due to whim or for the wrong or unsound reasons” (18). The court in Dasuki v Federal Republic of Nigeria (2016), adopted a definition of “arbitrary” as follows: “(1) depending on individual discretion; determined by a judge rather than by fixed rules, procedures or law; (2) of a judicial decision founded on prejudice or preference rather than on reason on fact.” In all the cases that have assumed the position of suspecting subjective claims of insanity, no reasons have been provided for substantiating the stand. It appears rather prejudicial to an accused person who otherwise should be entitled to a particular line of defence in the interest of a fair hearing since the plea is taken *cum grano salis*. The right to a fair hearing is guaranteed under Section 36(4) of the Nigerian constitution (Constitution of the Federal Republic of Nigeria, 1999).

More importantly, mental disorders constitute phenomenological issues primarily since they are essentially ‘lived experience’. Phenomenology is the study of structures of consciousness as experienced from the first-person point of view (19–21). The patient is the one who “owns” the experience and is thus an expert by experience (22, 23). Parties who are external to the experience, including the courts, then need to make sense of the phenomenon which the patient is trying to make sense of. This generates the “double hermeneutic” position in interpretative phenomenological analysis (23). Where this first-person experience is not taken into account, it may give rise to what has been termed “epistemic injustice” (24, 25). There are broadly two forms of this: testimonial and hermeneutical. In testimonial epistemic injustice, the words or knowledge of the individual is granted little credibility – the so-called credibility deflation (26) – while in hermeneutical epistemic injustice, the individual is impaired in their capacity to make sense of their own experience(s). Perhaps a form of testimonial epistemic injustice that is more analogous to the judicial view being addressed in this paper is pre-emptive testimonial injustice which precludes testimony due to a presumption of irrelevance or immateriality by those who have epistemic privilege and authority (27, 28) including judges.

On the basis of the foregoing formulation, this paper argues that Nigerian courts rely with greater confidence on non-expert fact-based accounts of defendants’ apparent behavioural abnormalities and familial vulnerability to mental illness (fairly objective historical facts) rather than their plausible phenomenological experiences (largely subjective contemporaneous facts) in reaching a conclusion of insanity. This is against a background of judicial neglect of medical opinion in insanity cases based on the notion that the determination of insanity in the legal sense is the sole preserve of the courts. Research has shown that expert opinion is sought in a little over a third of reported criminal cases with the insanity plea being mainly utilized in murder trials (12). Yet the experience of ‘mental disease’ or ‘natural mental infirmity’ as construed in the insanity plea falls in the realm of psychiatry which is a well-recognized subspecialty in modern medicine (29–32).

The inquiry addressed by this paper is relevant to the practice of forensic psychiatry and psychology for a number of reasons. In its focus on interrogating the application of legal tests for insanity, it has implication for the education and training of expert psychiatric witnesses as well as the development of forensic psychiatric practice in Nigeria. This review similarly highlights the importance of utilizing expert witnesses by the courts especially in cases where there is only a subjective claim of psychiatric illness. This paper also has psycho-legal implications as well. From a fundamental research and reform-focused perspective (33, 34), it is our opinion that the current judicial approach of discounting the defendant’s subjective experience, particularly in the presence of a credible expert opinion, is not in the interest of justice and should stop being the default mode of the final arbiters in the determination of legal insanity. Fundamental legal research aims to gain a deeper understanding of the law as a social phenomenon that has an impact on multiple disciplines, in contrast to reform-oriented legal research, which examines legal rules and highlights areas of inadequacy (34). The Fundamental legal approach recognizes the multidisciplinary utility in law which in this case is the intersection between psychiatry, psychology and the law. Relevant references to the legal implications of diagnoses, neuroscience data, and psychological assessments are made in order to highlight this crucial interface within the article.

The conceptual analysis in this paper is underpinned by two legal theories: the theory of legal positivism and the legal realism theory. Legal positivists seem to agree that the validity of a norm in any legal system is solely based on its endorsement, invocation, practice or enforcement by some relevant agents at a relevant time (35). Thus, the validity of the law is not based on its merit but on its existence and sources. This source-merit dichotomy has been framed in two central theses: (i) the social thesis, and (ii) the separability thesis. The social thesis postulates that law is mainly social fact or convention while the separability thesis establishes the notion that law and morality are separate (36) although this is not without dissent (37–39). Against this background, positivists uphold the merit of legal precedents as the position of the law. As Gardner argues: “the judge-made law…is legally valid because some judge or judges at some relevant time and place announced it, practised it, invoked it, enforced it, accepted it, or otherwise engaged with it.” In the current study, legal positivism has an explanatory function toward our understanding of the established habitual judicial disregard for uncorroborated subjective claims of insanity in Nigerian courts. This is framed on the basis that some judges in criminal cases have been observed to unquestioningly adopt the obiter (i.e., comments made in passing by a judge on a matter rather than the actual rule of law upon which a judicial decision is made) established by judicial discretion in Guobodia v The State as a valid precedent.

On the other hand, legal realism (40) holds the position that judicial acts were not impersonal or mechanistic but infused with personal values, political leanings and ideological preferences of judges. Legal realism argues that judges make decisions by “feelings” and “hunches” and then provide deliberative reasoning which will justify those decisions (41). It has been suggested that legal realism seeks to accommodate three tensions: between reason and power, science and craft, and between tradition and progress (42). In the current study, legal realism aids our understanding of the possibility of arbitrariness and inherent bias that could underline the judicial discretion established against uncorroborated subjective claims of insanity in Nigerian courts.

This paper presents its arguments using illustrative cases as well as a conceptual critique of judicial reasoning in decided insanity cases. The rest of the paper is arranged as follows: section two conducts a brief review of the Nigerian insanity plea with applicable rules of evidence while section three offers a critical analysis of illustrative cases involving the insanity plea.Section four focuses on a summary of the South African legal position on the insanity defence for the purpose of jurisdictional comparisons. Section five presents the discussion of the findings while section six provides a recommended model for judicial decision-making in relevant instances and conclusions are presented in section seven.

## 2. A brief review of the Nigerian insanity plea and applicable evidentiary rules

The Nigerian insanity defence is composed of two parts (Laws of the Federation of Nigeria, 2004a). The first limb serves as an exoneration by recognising that mental illness or a natural mental infirmity can impair one’s ability to understand, control, or recognise the wrongness of behaviour. A second limb is non-exculpatory in that it holds the defendant accountable for the extent to which particular delusions cause him or her to act legally or illegally. Despite this, the M’Naghten’s laws (Penal Code Law, 1959) are largely preserved by the Penal Code Act, Section 51 (Northern Nigeria), and it lacks a volitional component.

For emphasis, a critical ingredient in the Nigerian insanity plea is the presence of a ‘mental disease’ or ‘natural mental infirmity’ as the basis for lack of capacity in certain cognitive and volitional domains. In Rex v Ashigifuwo (1948), the court was inclined to recognize a disordered state of the mind as a spectrum stretching from ‘disease of the mind’ to ‘natural mental infirmity’. Fortunately, natural mental infirmity has been fairly defined as: *“a defect in mental power neither produced by his own default nor the result of the disease of the mind”* (Rex v Omoni, 1949). A similar dimensional perspective has been adopted in Ghana since the second half of the 20th century (43, 44). Conceptually, “defect in mental power” would most closely resemble intellectual disability or various forms of neurodevelopmental disorders such as autism spectrum disorders, specific learning disorder, and attention deficit hyperactivity disorder which may be associated with impairments in intellectual functioning (5, 10). Intellectual disability (intellectual developmental disorder) reflects deficits in general mental abilities including reasoning, abstract thinking, problem solving, academic learning and learning from experience.

From a biomedical perspective, the two variants of mental abnormality imply the presence of recognizable mental disorders and is a point of science upon which expert opinion ought to be sought (Evidence Act, 2011; (2)). In spite of this, the insanity defence suffers from the problems of its inconsistent public perception as well as discrepancies in medical testimony regarding the same defendant across the adversarial parties (2, 45).

The burden of proof with regard to insanity cases has been extensively addressed by the Evidence Act (2011). Section 136 provides that the burden of proof regarding any fact (e.g., insanity) is on the party which seeks to convince the court as to the existence of that fact. This provision may also ensure that the party adducing such evidence is allowed to prevent the opposing party from adducing evidence on any other relevant matter (section 138). While section 135 places a burden of proof beyond reasonable doubt on the prosecution in criminal matters, this shifts once the defence raises any exception to criminal liability as afforded by section 139(1). Where the insanity defence is thus raised as an exemption to the criminal conduct, section 137 places the burden of proof on the balance of probability on the defendant. However, section 138 of the Evidence Act (2011) grants the court wide powers with regard to the existence of facts related to the admissibility of any evidence by indicating that the existence or non-existence of those facts is to be determined by the courts.

It is here submitted that this determination becomes an opinion of the court. With regard to insanity, this opinion is formed on a matter that is largely one of science and not merely one of every-day facts. The Evidence Act (2011) provides guidance in respect of such opinions. Section 68 stipulates that persons who have special skills in certain foreign laws, customs, science or art are regarded as “experts” and their opinions in such specialized matters aid the courts in framing legal decisions. An expert is defined by a combination of knowledge, experience and skill (46–48).

Unfortunately, the courts are not compelled by this act to call expert evidence. Research evidence suggests that expert opinion is sought in slightly over a third of reported criminal cases which are murder trials (12). Its rate of success is about 26.5% and plea success is not associated with the utilisation of expert opinion. Significant correlates of a successful insanity plea have been found to be the use of limb one (especially inability to understand action/omission or control oneself) and unfamiliarity with the victim (12). The lack of obligation on the court to call expert witnesses in cases where the insanity plea is raised is in stark contrast to a comparable jurisdiction such as South-Africa where the court is obliged to call upon such experts, as will be explained in section four of this contribution.

## 3. Critique of illustrative cases

### 3.1. Guobadia v state (2004)

This was an appeal case in which a man was charged with the murder of his 2-year-old step-brother. His plea of insanity was made on the basis of a strange experience of being in ‘dream land’ and being ‘pursued by someone’. He thereafter ran into ‘something’ and found that he had stabbed the two-year-old. The appellant’s father testified for the prosecution and indicated that the defendant had a history of mental illness treated by traditional healers (previous act). The psychiatrist who saw the accused in custody (1 year and 2 months after the crime) found no evidence of mental disorder. However, the investigating police officer who took the defendant’s statement observed that the behaviour of the appellant was ‘abnormal’ (contemporaneous act). The trial court disbelieved the appellant’s testimony that he did not know what he was doing. Subsequently, the Supreme court held as follows: *“evidence of insanity tendered by an accused person himself is suspect and is not usually taken seriously.”*

In this instance, a history of mental illness existed although not treated by orthodox means. The fact that it was not treated in an orthodox setting does not negate its existence since the Supreme court had not indicated that treatment in orthodox settings alone constituted validation of a history of mental illness. This gives rise to a *previous* act as required in *Madjemu*. The explanatory power of this previous act with its need for intervention (traditional healing) is that it antedated the crime suggesting its role in affording the accused the legal excuse of insanity. An objective observation of his *contemporaneous* abnormal acts not long after the crime by the investigating police officer provides another acceptable criterion. However, the medical opinion negated the subjective experience of the appellant perhaps suggesting that he had no symptoms at the period of the psychiatric consultation and at the time of the offence. The failure of his defence under limb one of the insanity plea (Laws of the Federation of Nigeria, 2004a) is not the bone of contention since the court has the final say in the determination of legal insanity.

However, the *obiter*^1^ offered by the Supreme court in stating that the insanity claim should be “suspect” and not to be “taken seriously” bears careful attention. Indeed, in a transient departure during the judgement in the court of first instance, the learned judge held the view of giving the accused the benefit of his claim by stating thus: “Assuming for the sake of argument that the story he has told is true…” This was not only fair but consistent with the insanity criteria espoused by the Supreme court previously in providing guidance on the determination of legal insanity. The trial court, in its wisdom, then proceeded to find that the story even if believed could not justify the killing. In arriving at the conclusion that the story was not believable, it would appear that the balance of probability was not resolved in favour of the defendant. This is not particularly consistent with the legal principle of resolving doubt in favour of an accused person in criminal cases (Anubalu v. State, 2019). Additionally, in deciding that the “abnormal behaviour” of the accused observed by the investigating police officer was “of no moment,” the Supreme court on appeal also appeared to have trivialised the phenomenological possibility (or even plausibility and/or probability) of mental distress even though this decision was rightly framed within the overall context of legal insanity rather than merely a speculation on the existence or otherwise of mental disorder.

### 3.2. Saidi Oseni v. the state (2017)

This was an appeal case in which the defendant killed a woman by striking her with a cutlass. He had strange beliefs in which he claimed that his supposed lover was being hidden from him by the woman. He claimed he felt he was ‘possessed’ by a spirit on the day of the offence and believed he was under a spell cast upon him by a witch (Contemporaneous acts) and this led to his attacking the woman. The overall opinion of the expert^2^ was that the patient, at the time of the killing, was suffering from symptoms of paranoid schizophrenia^3^ which probably robbed him of the capacity of knowing that what he did was wrong as well as the capacity to control himself. The history obtained from the defendant himself revealed that he had previously been treated for a mental disorder in a private hospital but no records were available. Based on the claims of the defendant, a social worker visited his village and obtained a family history of mental illness in the mother.

In spite of the above, the trial court held that the defendant was not insane in the legal sense for the following reasons: (i) accounts of the abnormal state of mind came from the defendant himself (‘suspect’ and ‘not taken seriously’ per *Goubodia*); (ii) the opinion of the expert witness could not be relied upon since there was no ‘conclusive diagnosis’ of insanity; (iii) the history of mental illness in the mother not backed up by ‘scientific’ or ‘medical’ analysis and that neither the mother of the patient nor the social worker who got the history of mental illness in the mother testified in the case; (iv) there was no cogent evidence of the past mental state of the accused person before the alleged incident.

However, the appeal court held that the burden of proof with regard to insanity was a light one and one to be discharged on the balance of probability or preponderance of evidence. It was not to be viewed speculatively or *via* tenuous inference but on the basis of the evidence before the court. The court further held that the proof of insanity could be constituted by “any or a combination” of the criteria outlined by the Supreme court as evidence supportive of insanity (see section 1.0 above). The list not *qua* conclusive, the court might equally rely on any other relevant fact which may assist it in coming to a conclusion of legal insanity. Additionally, the burden of proof of insanity was interpreted carefully as not one “beyond reasonable doubt.” Once the accused had led relevant probable/preponderant evidence in support of his claim, the burden was shifted to the prosecution to rebut the claims of the accused. If the resultant burden on the prosecution was not discharged, the claim of insanity should succeed.

The court declared that to require more from the accused would be to demand proof of innocence or sanity which are constitutionally and statutorily presumed (Constitution of the Federal Republic of Nigeria, 1999; Laws of the Federation of Nigeria, 2004a). The court cautioned that while the trial court had the discretion to believe or disbelieve the testimony of the medical expert, the decision to reject expert opinion ought to be supported by a “reasonable hypothesis derived from the evidence on record.” Based on the failure of the prosecution to controvert the evidence of insanity (subjective claims supported by expert opinion), the initial finding of guilt was reversed on appeal and a verdict of “Not Guilty By Reason of Insanity” was substituted with the accused remanded in an asylum during the pleasure of the Governor.

## 4. Exploring an alternative position: The insanity defence and expert opinion in South African courts

The South-African legal framework that regulates the insanity defence, also has its roots in the M’Naghten rule (51) as the case is in Nigeria. The South African Criminal Procedure Act 51 of 1977 (CPA) cements the presumption of sanity in that section 78(1A) states that every person shall be presumed not to suffer from a mental illness until the contrary is proven. The test for lack of criminal responsibility is set out in section 78(1) of the CPA which recognises that “mental illness” or “intellectual disability” may render an accused incapable of appreciating the wrongfulness of his conduct or acting in accordance with an appreciation of wrongfulness.

The burden of proof regarding the accused’s criminal liability rests on the party who raised the issue (52). The presence of mental illness or intellectual disability has to be proved on a balance of probabilities (53). Thus, if the accused avers that he is not guilty on account of mental illness (the insanity plea), then he needs to prove the presence of a mental illness at the time when the crime was committed.

It was pointed out earlier that Nigerian courts do not attach a lot of weight to the personal account of an accused who alleges that he had a mental illness when the crime was committed. The South African position is that a court must refer such an accused for a forensic assessment by a court appointed mental health professional or panel of such professionals (CPA s78(2)). Where it appeared from the accused’s general demeanour in court and his interactions with his legal representative that mental abnormalities may be inferred, the court ought to proceed in terms of s 78(2) (S v Mphela, 1994; (53)). Snyman warns, however, that the fact that an accused has a bizarre defence against a charge, does not necessarily imply that the person has mental health problems that may impact his triability (54).

Thus, it is not left up to the court to decide if the “alleged” presence of a mental illness is real or credible. This is determined by experts in the field with the necessary knowledge and experience. In S v Mabena (2007), Nugent JA observed as follows:

*“Mental illness” and “mental defect” are morbid disorders that are not capable of being diagnosed by a lay court without the guidance of expert psychiatric evidence. An inquiry into the mental state of an accused person that is embarked upon without such guidance is bound to be directionless and futile.”*

Once it is decided that the accused must be assessed forensically for criminal capacity, the court appoints one psychiatrist to do the assessment if the charge against the accused is of a minor nature (s 79(1)(a) of the CPA). For more serious offences, the court appoints a panel of experts consisting of the head of the psychiatric hospital or a psychiatrist appointed by him or her, two further psychiatrists (one appointed by the court and one by the accused) and a clinical psychologist where the court so directs (55). The psychiatric interview *inter alia* focuses on the individual’s account of the crime (56). The accused is thus granted an opportunity to convey his personal account of events and how s/he experienced it to a qualified mental health professional. Pillay (57) adds that the assessment further entails interviews with the accused and his family members.

The mental health professionals must provide a report to the court setting out the diagnosis (s 79(4)(b) of the CPA) and a recommendation on whether the accused was criminally responsible at the time of the commission of the offence or not (s 79(4)(d) of the CPA). Where the finding is not unanimous, this must be stated in the report and the differing findings must be stated. (s 79(5) of the CPA).

If the report is unanimous, the court may determine the matter based on that report without hearing evidence on it – provided the prosecutor and the accused do not dispute it (s 79(3) of the CPA; S v Sithole, 2005; S v Vika, 2014). If the report is not unanimous, the court may hear evidence including from those who conducted the psychiatric assessment under section 79. The same applies if the accused or prosecutor disputes the findings in the report (De Vos N.O and Another v Minister of Justice And Constitutional Development and Others; In Re: Snyders and Another v Minister of Justice And Constitutional Development and Others, 2014).

If the recommendation is that the accused was indeed criminally responsible but that his ability to appreciate the wrongfulness of his act or to act in accordance with such appreciation was diminished, such diminished capacity will be taken into account during sentencing per section 78(7) of the CPA (see also: S v Romer, 2011; Ngobeni v The State, 2018). Factors like mental illness, provocation, jealousy, severe emotional stress or even intoxication may affect the offender’s emotions to such an extent that his/her criminal responsibility may be diminished. Diminished criminal responsibility presupposes criminal capacity but reduces culpability. Diminished criminal responsibility is not a defence against a charge (as the case is with a lack of criminal capacity due to mental illness or intellectual disability) but rather a factor to be considered during sentencing. (S v Mnisi 2009 (2) SACR 227 (SCA) at para 4).

Furthermore, diminished criminal responsibility allows for the impact of perhaps a less serious mental illness to be taken into account when delivering a sentence in cases where the mental illness did not have a “serious enough” impact on the accused’s criminal capacity to render him/her not criminally responsible. Cases where diminished criminal capacity is alleged present an exception to the rule in the South African criminal justice system that mental health professionals should be approached for a forensic assessment in cases involving mental illness. This exception may be because diminished criminal capacity presupposes criminal capacity. The court may, however, consider expert evidence (if available) in mitigation of the sentence.

The above process allows an accused to convey his personal experience of how the mental illness impacts him/her to a mental health professional with the necessary skills and experience to interpret such information. The court’s initial “suspicion” that an accused might have a mental illness may very well be confirmed through this process. Such an enquiry may also clear up the position for the court in cases where the court was doubtful of the authenticity of the display of what seemed like symptoms of a mental illness. The burden is not placed on the court to decide whether the accused has a mental illness or intellectual disability, this decision is made by mental health professionals, as it should be.

The final decision concerning the criminal capacity of the accused is a legal one and is ultimately taken by the court (58). This legal decision determines future legal proceedings against the accused in that a finding of lack of criminal capacity might lead to an acquittal. In contrast, a finding that the accused had criminal capacity will cause the criminal trial to proceed without further consideration of the alleged mental illness (unless it is raised during the sentencing phase as a mitigating factor, for example, diminished criminal responsibility). The court in South Africa is, however, always guided by expert opinion in cases in which criminal capacity could be impacted by mental illness as it acknowledges that it is not an expert in the field of psychiatry and will generally not deviate from the recommendations made by the mental health professional (S v McBride, 1979; (53)). Where the court ignores the rule that an expert should be consulted whenever the effect of the accused person’s mental state on criminal capacity is at issue, and the court finds such an accused not criminally responsible, that judgment could be taken on appeal (S v Magongo, 1987).

From the above, it is clear that South African courts are not left to their own devices when deciding whether a mentally disordered accused is criminally responsible. Whenever the court suspects that an accused may have a mental illness that impacts his/her criminal capacity, its presence or otherwise must be determined by mental health professionals. This position ensures that expert opinion is duly considered in cases involving mental illness, which aids in the delivery of justice.

## 5. Discussion

The aim of this paper was to demonstrate the misalignment between the subjective experience of the mentally ill defendant and the objective judicial unawareness of their internal states in certain cases. This discrepancy could potentially lead to a miscarriage of justice as shown by one case reversed on appeal. The implications of our findings for the practice of forensic psychiatry and psychology are numerous. First, this paper has broadened our understanding of the application of legal rules relevant to the insanity plea in Nigeria (Laws of the Federation of Nigeria, 2004a; Penal Code Law, 1959) and seeks to refine such applications (11, 59, 60). The evolution and/or refining of legal tests for insanity has been crucial to the development of expert witness roles in psychiatry and psychology over time (1, 3, 4, 47, 61). Indeed, the search for relatively objective evidence in insanity cases by courts all over the world in the interest of justice and fairness has led to the use of psychiatric, psychological, neuroscience and other relevant forms of scientific evidence in the court room with appropriate safeguards (62–67).

Second, this paper contributes to expert witness education in reminding the expert witness that their clinical assessments seek to answer critical legal questions which have direct bearing on justice and the culpability or otherwise of patient-defendants (48, 68). In so doing, the psychiatrist is reminded that the ultimate issue is one for the court to decide (Evidence Act, 2011; (12, 47)) but great direction may be obtained from well-framed expert opinions based on the available evidence which may sometimes be unreliable in the eyes of the court. In this connection, a lack of rigor on the part of the expert witness may inadvertently contribute to invalidating the subjective plausible experience of the defendant which the court is already hard-pressed to reject.

Third, this review could potentially educate judicial actors in Nigeria on the need to rely appropriately on the assistance of credible expert witnesses in making non-dispositive determination of the existence of mental disorder in defendants within the overall context of the adjudication of insanity cases (2, 43, 69). Expert witnesses have a more crucial role to play in cases where the defendant only has subjective claims of mental illness as the court would benefit significantly from honest, neutral and relatively objective opinion evidence as is ethically required of experts (64, 69–71). Fourth, the disposal of insanity cases has implication for the practice of forensic mental health both within the secure forensic care context and prison mental health care in terms of the assessment and treatment of those adjudged as mentally disordered offenders. Undue criminalization of the mentally ill has significant justice and quality of life implications for such defendants when imprisoned and also poses clinical as well as ethical problems to practitioners and society (72–74).

A most significant issue raised by our analysis is the fact that symptoms of mental disorder are largely phenomenological – first-person in nature (24, 75–77). For instance, research has shown that there are activations in both speech and auditory areas in the brains of individuals with schizophrenia experiencing auditory hallucinations (78). These voices are all in their brains and not available to others apart from themselves. Notwithstanding this, such brain abnormalities while serving limited explanatory function for the underlying mechanism of developing subjective symptoms, may not necessarily generalize to all individual psychotic experiences. There are also the challenges of first-person data in cognitive neuroscience which include bias and inaccuracy. Indeed, the process of generating first-person report may modify the experience as well as produce an ‘explanatory gap’ in relating first-person data (‘I’) to third-person behaviour (‘He’) (79). The objective interpretation of an individual’s subjective experience based on observable disposition by second or third parties may not always be accurate and to hold the objective as superior to the subjective experience or its autologous explanation could result in hermeneutical epistemic injustice (24). Cognitive biases such as those related to monocausal attribution and jumping to conclusion may be found in those with schizophrenia and other non-affective psychoses and such biases could potentially provide the basis for delusional ideas which patients may act upon (80–84). Nevertheless, it is vital to note that these biases are not specific to those who have psychosis and may not singularly cause delusional states.

Beyond the subjectivity of psychiatric symptoms however, it is important to address the existence of objective psychiatric and psychological assessments of alleged mentally ill defendants. These are admittedly often based on client self-report but go beyond the subjective by treating speech acts, empirically based psychometric measures, and motor behaviors which are objectively observable and measurable. Such assessments include scores on cognitive tests and personality inventories as well as findings on mental state examination (85, 86) even though they have recognised limitations (87). Additionally, unobtrusive observation of patients during hospitalisation may also yield objective verifiable information beyond subjective claims (88). These approaches reflect neither purely defendants’ subjective information nor entirely expert “opinion” (since they are reproducible across practitioners) and may complement subjective reports to form a basis for diagnosis and psycho-legal formulation preparatory to forming an expert opinion.

In particular, there are tools that could assist in assessing the likelihood of malingering (13, 89, 90). These will undoubtedly address some of the concerns raised by the courts regarding the unreliability of subjective claims of insanity which have the potential of being feigned. In the Nigerian context, some of these tests are not frequently used (88) and this review highlights the need for more frequent involvement of such objective measures in the assessment of criminal defendants claiming insanity.

It is reasonable to concede that where this subjective experience of mental symptoms is the sole ground for an insanity defence, the court ought to entertain the danger of deception. Even then, it is better to err on the side of justice by resolving the doubt in favour of the accused except in instances where other relevant circumstances would lead to a different conclusion. However, where corroborative expert opinion is sought and obtained, the mere fact that the claim of symptoms is from the accused alone should not weaken its credibility or overall merit in the determination of the presence of clinical mental disorder and/or legal insanity.

While legal insanity is a legal determination, the central theme in its formulation is whether a mental disorder exists in the first place. Medico-legal debates continue as to the definition of insanity especially with regard to insane delusions (83). For instance, the legal tendency to regard insane delusions as being circumscribed and based on rational reasoning alone without recourse to emotion or impaired volition has been questioned by scientific findings suggesting that delusions are related to cognitive biases which impair moral decision-making (83). On the side of psychiatry, a more biomedical viewpoint on insanity is embraced by psychiatrists as physicians (30, 31). On this basis, mental disorders are diagnosed based on clinical signs and symptoms described in diagnostic manuals (10, 91) and supported by extensive clinical as well as laboratory investigations as required. To that extent, ‘Mental disease’ or ‘natural mental infirmity’ should be judgements in which the courts should defer to the medical expert while legal insanity based on the effects of these mental states on the requisite capacities should be determined by the courts.

This clearly raises the question of the position of the psychiatric expert with regard to the ultimate issue which in this case refers to whether the mental disorder deprived the defendant of the capacity to understand conduct, control action or appreciate the wrongfulness of act/omission. Jurisdictional variations exist as to whether or not the expert should address the ultimate issue (47). In Nigerian law, the Evidence act (2011) indicates a position of flexibility in which the expert may present an opinion on the ultimate issue although the conclusive finding on the issue is the sole preserve of the court. This is a tenable position and satisfies the quest of the judiciary to remain the final arbiter in judicial proceedings.

This paper also contributes to the legal literature and policy in a number of significant ways. First, to the knowledge of the authors, it is the first to interrogate the settled but non-evidence-based judicial inclination towards discountenancing subjective insanity claims even when consistent with psychiatric expert opinion. It adopts both reform-oriented and fundamental research approaches (33, 34) in order to achieve this objective. Without such inquiries, the mentally ill are unlikely to reap the benefits of therapeutic jurisprudence which is a humane approach of society to the treatment of the offender (92).

Second, it contributes to judicial policy by proposing a conceptual model that is supported by case law and contemporary exemplification for dealing with instances in which only the accused gives factual evidence as to their experience of psychiatric symptoms and when this evidence is validated by medical opinion. The premise of the current argument is that the courts ought to defer to the accused as an “expert by experience” as well as to objective expert opinion regarding the presence of mental disorder unless rebutted or discredited (see Saidi Oseni v. The State, 2017). Such an approach shows respect for the epistemic reality of the patient and avoids testimonial and hermeneutical epistemic injustice (25–28). This fair recognition of the defendant, in our view, does not injure justice or the logic of consistency in judicial reasoning since the ultimate question as to whether the mental disorder resulted in the criminal conduct is still left to the court as the final arbiter on legal insanity.

Third, it contributes to the utility of legal theory in relation to the insanity defence by interrogating the stance of legal positivism as well as legal realism in the judicial construction of insanity in Nigerian courts. On the one hand, legal positivists would argue that judicial suspicion of uncorroborated subjective claims of insanity represents the precedential position (35, 36) and it is consistent with the societal need to ensure that the threshold for legal excuses for crimes is not set too low. Additionally, the view that mental disorder should be so observable to others as to be unreliable when disclosed by the sufferer alone could be plausibly driven by “collective social imagination” (25) on the basis of long-held negative stereotypes and prejudices (26) towards the mentally ill.

On the other hand, legal realism suggests that this judicial position is not one arrived at on the basis of objective and mechanistic logical processes but one that could have been informed by judges’ personal prejudices (41) towards those who adopt such excuses based on the aforementioned collective social imagination. The harm done to justice and the constitutional right of the defendant in an attempt to preserve judicial discretion and reasoning latitude that may be framed by individual biases is one that commands crucial consideration. A realist orientation will aid judges in seeing the need to provide reasons for their refusal to accept subjective claims of insanity and help them to be more critically aware of their own biases or prejudices regarding mental illness which may unconsciously influence the exercise of their judicial discretion.

Perhaps another important psycho-legal issue to consider is that the pronouncement by the court of appeal in *Saidi Oseni* gives expert witnesses and judicial actors a sense of what might constitute the “balance of probability or preponderance of evidence” regarding the presence of insanity in the accused. Given the lightness of the burden of proof in insanity cases, the court has provided guidance in the frame that “any or a combination” of the outlined evidentiary criteria supportive of insanity may be regarded by the court as satisfying the burden of proof. Yet within this space of probative value, there is a tendency to discountenance the subjective experience of the defendant-patient based on the sometimes erroneous belief that mental illness would have been observable to third parties prior to the offence. This may not always be the case especially with first episode psychosis in which other people might not have particularly noticed aberrant behaviour in the defendant for a substantial period of time. Since the burden of proof for insanity cases is on a balance of probability, criminal courts need not impose a heavier burden on the mentally ill by requiring the ‘scientific’ or ‘medical’ analysis of the history of insanity (as suggested by the trial judge in *Saidi Oseni* in relation to familial history). Such a requirement seems to be an unduly high confirmatory threshold and tellingly introduces the bias instituted in *Goubodia* and related cases against a simplified (not simplistic) view of what constitutes a legal test for the presence of mental disease or natural mental infirmity.

## 6. Recommendations on adjudicatory models in insanity cases

The current formulation of legal insanity in Nigerian courts indicates that insanity is conceived by the judiciary as a legal opinion in its entirety and medical opinion is typically treated as one of the facts presented as evidence before the court in support of the claim. This resembles an omnibus approach. The recommended decision-making model is onewhich describes a three-stage algorithmic model which starts with entertaining expert opinion on the ground of fact and science (the doctor is the expert by knowledge and skill while the defendant-patient is the “expert by experience”). The second stage involves determining the effect of the disorder on cognitive or volitional capacity. The last stage is the adjudicatory phase in which the judge makes a ruling on culpability.

Based on the foregoing, the following recommendations are made:

- The courts ought to take the subjective uncorroborated claims of insanity (especially when supported with expert opinion) seriously and make certain that all necessary steps are taken to ensure that the claim is properly heard and examined. To refuse to attach any seriousness to it is to start the defence off on a platform of negative bias or prejudice. The attendant incredulity of the court may equally be extended to the opinion of the expert since a major portion of the substance of the expert’s opinion (self-reported symptoms of mental illness) was not to be taken seriously in the first place. In instances where expert evidence is called and is regarded as credible, the courts should hold such opinions as valid for the determination of the presence of “mental disease or natural mental infirmity” strictly. This, not being the ultimate issue, does not detract from the powers of the court to adjudicate independently.
- Currently, Nigerian courts typically request one expert witness who may be called by the defence or prosecution. As was the case in *Saidi Oseni*, the trial court disagreed with the only expert as to the conclusiveness of mental illness. In this way, judicial reasoning was substituted for expert knowledge and skill. To avoid this, it may be suggested that Nigerian courts should consider the possibility of allowing one expert on each side such that the court can exercise its discretion as to which expert to believe when experts disagree (62, 93). In this way, the court refrains from substituting judicial logic for medical opinion in matters in which the medical man is the expert. That said, the challenges to this alternative recommendation are human resource constraints and cost (47).
- Lastly, the South-African position could be adopted in that the court ought, in all cases involving allegations of mental illness, to appoint a mental health professional or a panel of such professionals, depending on the seriousness of the charges against the accused, to assess the accused. Such assessment will include a physical assessment and interviews with the accused to ascertain his subjective experience. The mental health professional(s) submit(s) a report to the court with their recommendations. The court remains the final decision maker but is guided by experts in the field. South African courts rarely deviate from the recommendations in the mental health professional’s report as to the presence or otherwise of mental illness, illustrating that the court acknowledges that it lacks expert knowledge on the topic and has confidence in medical opinion.

## 7. Conclusion

Overall, Nigerian courts currently place significant weight on non-expert accounts of defendants’ apparent behavioural disorder and familial vulnerability to mental illness as findings of fact and history. Plausible phenomenological experiences volunteered by defendants are regarded as generally suspect. This is not consistent with a valid phenomenological view of mental disorder and could eventually perpetuate epistemic as well as actual injustice. Thus, discounting the subjective experience of the defendant particularly in the presence of sustainable expert opinion could lead to miscarriage of justice and this judicial posturing should be reformed on the basis of current knowledge and exemplary developments in comparable commonwealth jurisdictions such as South Africa. To further assist itself in the transparent dispensation of justice, the Nigerian judiciary ought to more readily require the services of psychiatric expert witnesses especially in cases where non-expert corroborative evidence for insanity is lacking. Within this context, there should be greater emphasis on expert witness training for psychiatrists who are medically qualified to provide psychiatric expert opinions in Nigeria in order to enhance the credibility and reliability of such opinions presented to the courts in the process of adjudicating cases involving the insanity plea.

### Cases cited

#### Nigeria

Anubalu v. State (2019) LPELR 48088 (CA).
Dasuki v Federal Republic of Nigeria (2016) (ECW/CCJ/JUD/23/16) ECOWASCJ 54.
Emeryl v State (1973).
Guobadia v State (2004) 6 NWLR 360.
Madjemu v State (200a) (Pt. 52) FWLR 2210.
Rex v Ashigifuwo (1948) 389. W.A.C.A.
Rex v Omoni (1949) 12 W.A.C.A 511–513.
Saidi Oseni v. The State (2017) LCN/10139(CA).

#### South Africa

De Vos N.O and Another v Minister of Justice And Constitutional Development and Others; InRe: Snyders and Another v Minister of Justice And Constitutional Development and Others 2015 (1) SACR 18 (WCC).
Ngobeni v The State (A684/16) [2018] ZAGPPHC 715 (23 February 2018).
S v Mabena 2007 (1) SACR 482 (SCA).
S v Magongo 1987 (3) SA 519 (A).
*S* v *McBride* 1979 (4) SA 313 (W).
S v Mnisi 2009 (2) SACR 227 (SCA).
S v Mphela 1994 (1) SACR 488 (A).
S v Romer 2011 (2) SACR 153 (SCA).
S v Sithole 2005 (1) SACR 311 (W).
S v Vika (14519) [2014] ZAWCHC 155 (14 October 2014).

#### United Kingdom

M’Naghten’s case. (1843). UKHL J16.
R v M’Naghten (1843) E. R. (Vol. 8).

### Statutes

#### Nigeria

Constitution of the Federal Republic of Nigeria (1999).
Evidence Act (2011).
Laws of the Federation of Nigeria. *Criminal Code Act Chapter C39*., (2004).
Laws of the Federation of Nigeria (2004b). *Criminal Procedure Act, Cap C41*.
Laws of the Federation of Nigeria (2004c). *Interpretation Act. Cap 123*. Federal Ministry of Justice.
Penal Code Law (1959).

#### South Africa

Criminal Procedure Act 51 of 1977.

## Author contributions

AO conceptualised the study and wrote the first draft of the manuscript. LP contributed the section on the South African insanity jurisprudence. OO contributed to the write up of the final version of the manuscript and provided critical comments on scope. All authors contributed to the article and approved the submitted version.

## Funding

The authors thank Unisa for funding the processing fees of this article.

## Conflict of interest

The authors declare that the research was conducted in the absence of any commercial or financial relationships that could be construed as a potential conflict of interest.

## Publisher’s note

All claims expressed in this article are solely those of the authors and do not necessarily represent those of their affiliated organizations, or those of the publisher, the editors and the reviewers. Any product that may be evaluated in this article, or claim that may be made by its manufacturer, is not guaranteed or endorsed by the publisher.

## Footnotes

^1^While obiter comments ("obiter dictum" in full) are not legally binding on lower courts in guiding their legal decisions, they are quite persuasive and strongly instructive especially when regarded as "seriously considered" (49, 50).

^2^The first author was the expert in the case.

^3^Schizophrenia is a major mental disorder characterized by strange and usually false beliefs, hearing voices of unseen persons, seeing things unseen by others in clear consciousness, or experiencing undue external control of one’s actions/thoughts which may manifest with irrational speech, beliefs that one is being persecuted by others, among other things.
